# Life course epidemiology: Modeling educational attainment with administrative data

**DOI:** 10.1371/journal.pone.0188976

**Published:** 2017-12-27

**Authors:** Leslie L. Roos, Elizabeth Wall-Wieler

**Affiliations:** 1 Department of Community Health Sciences, University of Manitoba, Manitoba, Canada; 2 Manitoba Centre for Health Policy, Manitoba, Canada; Karolinska Institutet, SWEDEN

## Abstract

**Background:**

Understanding the processes across childhood and adolescence that affect later life inequalities depends on many variables for a large number of individuals measured over substantial time periods. Linkable administrative data were used to generate birth cohorts and to study pathways of inequity in childhood and early adolescence leading to differences in educational attainment. Advantages and disadvantages of using large administrative data bases for such research were highlighted.

**Methods:**

Children born in Manitoba, Canada between 1982 and 1995 were followed until age 19 (N = 89,763), with many time-invariant measures serving as controls. Five time-varying predictors of high school graduation—three social and two health—were modelled using logistic regression and a framework for examining predictors across the life course. For each time-varying predictor, six temporal patterns were tested: full, accumulation of risk, sensitive period, and three critical period models.

**Results:**

Predictors measured in early adolescence generated the highest odds ratios, suggesting the importance of adolescence. Full models provided the best fit for the three time-varying social measures. Residence in a low-income neighborhood was a particularly influential predictor of not graduating from high school. The transmission of risk across developmental periods was also highlighted; exposure in one period had significant implications for subsequent life stages.

**Conclusion:**

This study advances life course epidemiology, using administrative data to clarify the relationships among several measures of social behavior, cognitive development, and health. Analyses of temporal patterns can be useful in studying such other outcomes as educational achievement, teen pregnancy, and workforce participation.

## Introduction

Educational attainment has been linked with many neighborhood, family, and individual characteristics. Children living in low socioeconomic neighborhoods tend to have significantly lower levels of educational attainment.[1] Family variables, such as structure changes and mobility, also influence educational attainment. Children who experience family disruptions often have lower educational attainment due to the fewer resources and less time to monitor school work associated with single parent families.[2,3] Residential mobility, particularly if the child is in school, can also negatively affect educational attainment.[4] Children in poor families often move more than children in rich families, and are more likely to attend schools with high student mobility rates.[2] Childhood health can significantly affect educational attainment; health conditions can affect development, and children with health conditions may have to miss school more frequently. The most common reason for seeking medical treatment among children is injuries.[5] Mental health conditions can significantly reduce educational attainment, with externalizing disorders, such as Attention-Deficit/Hyperactivity Disorder (ADHD) and conduct disorders having the largest effect.[6]

As previous research indicates, inequalities in education and health that emerge in late adolescence and adulthood are often rooted in childhood or even in the fetal period.[7] Using a developmental perspective to examine such inequalities assesses important processes across the life course by focusing on the effects of physical or social exposures at several points in time.[8,9] Accounting for these processes depends on very large numbers of cases with many variables measured over substantial time periods. Such observational research has been restricted by the limited availability of datasets adequate to characterize life course trajectories and distinguish among various possible models.[9] Existing studies generally focus on the temporal pattern of influence of a single time-varying predictor.[10–12] Such work provides insight into the pathway of influence for that measure but does not address how pathways may be affected by other important variables. The linkable administrative databases being created across the developed world are uniquely valuable in assembling a wide range of temporal variables starting at birth and continuing well into adulthood.[13,14] At the same time, disadvantages associated with using these data need to be acknowledged. Examining several time-varying predictors simultaneously facilitates a more complete understanding of the dynamic effects of social and biological risk factors on later outcomes.

We used administrative data to generate birth cohorts and to study pathways of social and health inequality in childhood and early adolescence leading to differences in educational achievement.[15] Determining those periods in which specific events have more prominent effects on later life outcomes is crucial for life course epidemiology.[16] Researchers have proposed several conceptual frameworks for the different processes involved in the first two decades of life.[9] The ‘accumulation of risk’ model refers to the adverse effects on later health of exposures accumulating over the life course.[9] Sensitive period models postulate that exposures exert their greatest effects during particular times but are modifiable by other influences. Critical period models refer to “exposures acting during a critical window of development” affecting later development.[9] Work on the fetal origins of adult disease provides an important example of such a “critical period” approach.[17]

Operationalizing and testing different life course models requires data which are seldom available. This research examines the patterns by which five time-varying predictors—three social (Living in a Low Income Neighborhood, Residential Mobility, Family Structure Changes) and two health (Externalizing Mental Conditions, Injuries)—affect the odds of high school graduation. Given the importance of high school graduation on social and economic opportunities, gaining a better understanding of how and when specific events in childhood and adolescence affect these outcomes is necessary.[16] The following questions are addressed: Which measures best predict high school graduation? Do significant time-varying covariates operate in a cumulative fashion, in sensitive periods, or in critical periods? Do the temporal patterns selected for each time-varying covariate differ when adjusting for measured and unmeasured confounders?

## Materials and methods

### Data overview

The Manitoba Population Research Data Repository at the Manitoba Centre for Health Policy (MCHP) contains province-wide, routinely collected information over time for each family and for each resident. A research registry, created through linkages between the provincial health registry and Vital Statistics files, permits identifying when an individual first took up residence in the province, when he/she left, place of residence (postal code), deaths, births, and key familial characteristics.

Outcomes are modelled using a large sample of children born in Manitoba, Canada from 1982 to 1995 and staying in the province until their 19^th^ birthday. Manitoba is reasonably representative of Canada on a number of measures (health status, health care expenditures, and education) [18]. Data quality and loss to follow-up (approximately 23%) are discussed in S1 File and elsewhere.[18–20] The final cohort (n = 89,831) includes individuals with no missing data; approximately 9% of the cohort was missing information on at least one variable. The majority of those excluded due to missing data were only missing information on high school graduation. Many of the schools run by First Nations (Indian) bands in the Rural North do not report information to the Ministry of Education and Training; this seems responsible for most of the missing cases. Missing data are discussed more extensively elsewhere.[21] S2 File and S1 Fig describe cohort selection.

### Variables

#### Outcome

Failure to graduate from high school was defined as not finishing high school within four years of entering grade nine. Analyses of five-year graduation rates produced very similar results.

#### Covariates

Covariate selection is based both on data availability and on previously identified predictors of educational attainment.[2–4,16,22–29] Time invariant cohorts include health at birth, measured by birth weight, and socioeconomic status, measured using mother’s marital status at the time of birth and mother’s age at first birth. We also controlled for birth year, family size, birth order, sex, and urban/rural residence.[18,23] Graduation rates (which might reflect varying provincial socioeconomic circumstances in childhood) did not differ substantially with birth year. Earlier educational achievement was assessed using the Grade 9 Achievement Index, based on average marks in all classes and the number of credits earned during the school year.[21,30] Those doing better than the mean on this index received a 1 and those doing worse a 0. In previous work, these time-invariant measures have explained approximately as much variance in predicting educational outcomes as those in such well-known American research as the Panel Study of Income Dynamics (PSID).[21]

Events may be experienced at various stages of development; data for each time-varying predictor are available for three periods: ages 0–3 (preschool), ages 4–8 (early elementary school), and ages 9–13 (early adolescence).[23] Three social variables: Living in a Low-Income Neighborhood, Residential Mobility (measured by a change in six-digit postal code, which often corresponds with one city block) and Family Structure Changes (parental death, divorce, remarriage, marriage), and two health variables: externalizing mental health conditions (ADHD and/or CD/ODD) and injuries are examined. Two other time-variant health conditions (asthma and the number of major health conditions) were not included, given both their lack of importance in earlier research and preliminary runs using these data.[23]

Low-income neighborhoods are Canadian census areas with an average income falling within the lowest twenty percent of the distribution; to be noted as living in such a neighborhood, individuals had to reside in a low-income area for at least six months within a given time period. Canadian census areas are typically smaller than their American counterparts, with populations between 2,500 and 8,000 persons.[21,31] Living in a low-income Manitoba neighborhood has been shown to be highly correlated with low household income; using the more common household measure (on a somewhat different data set) changed results only slightly.[21,32] Residential mobility is defined by movement between 6-digit postal codes. Two or three postal codes are characteristically included in an urban census area. The presence of at least two diagnoses (hospital separations or medical abstracts) within a four-year time period defined externalizing mental health conditions (ICD-9 code 312, 313, 314; ICD-10 code F90, F91-F94, F98). Injuries are defined by at least one hospitalization within a four-year period having an external causes of injury code (ICD-9 codes beginning with the letter ‘E’, ICD-10 codes beginning with the letters ‘V, W, X, and Y’).[33] Approximately 98 per cent of Manitoba children will have seen a physician in a four year period. In line with earlier research, each health condition was ‘time-limited'; if an individual received a diagnosis for a specific condition in an earlier but not in a later age group, that individual would only be identified as having the condition at the earlier age.[23]

### Measurement

The model-building framework developed by Mishra et al. facilitates examining the magnitude and timing of social and health events in childhood and early adolescence on high school graduation.[15] For each time-varying exposure, six alternative hypotheses were tested: the full model, accumulation of risk, sensitive period, and three critical period hypotheses.

#### The full model

An individual experiencing time-varying covariate *S* in age group *i* (*i* = 1, 2, 3; 1 = Ages 0–3, 2 = Ages 4–8, 3 = Ages 9–13) received a value of ‘1’ for variable *S*_*i*_. Each time-varying covariate is represented as a combination of all *S*_*i*_. For example, if an individual only moved between ages 4–8, they would have a value of ‘1’ for *S*_*010*_ for ‘Residential Mobility’. Equation (A) is presented below:

$$\beta_{1}S_{100}+\beta_{2}S_{010}+\beta_{3}S_{001}+\beta_{4}S_{110}+\beta_{5}S_{101}+\beta_{6}S_{011}+\beta_{7}S_{111}$$ (1)

The full model is a logistic regression model with a set of background variables, as well as the seven temporal variables in Eq (1), for each of the five time-varying covariates.

#### Temporal patterns

The accumulation of risk model assumes that the longer an individual spends with a certain exposure, the greater the effect on the outcome, irrespective of when those exposures occurred.[15] Risk associated with exposure is assumed equal across the life course; experiencing an exposure in any two time periods generates twice the risk of an exposure in any one time period.[34] In a sensitive period model, risk still accumulates, but varies across developmental periods.[34] In a critical period model, the exposure of interest has an effect (protective or adverse) on the outcome only during a relatively short window of time; outside of this window, this mechanism does not exist.[34] Three critical period models are tested—the early childhood critical period model, the early elementary critical period model, and the early adolescent critical period model.

#### Trimming the full model

Mishra et al.’s framework “formally compares alternative hypotheses on the effect of multiple binary exposure measurements” over the first fourteen years of life.[15] Specific research questions include: does changing residence at any age have an effect? Does risk accumulate in a regular fashion? Is exposure in more than one age group significant in generating risk for not graduating (sensitive period model)? Does moving at just one age period affect the probability of graduation? Such hypotheses were tested against the full model using a likelihood ratio test to determine the best model for each time-varying covariate. A likelihood ratio test with a non-significant p-value (p > 0.05) indicates that the simpler model fits the data as well as the full model. The temporal pattern of each covariate was tested in the presence of the full model of the other covariates. The following steps were taken to identify the best temporal pattern for each time-varying covariate:

1. When a simpler model fits the data as well as the fill model, that model was selected.
2. If more than one nested model had a p-value greater than 0.05, the model with the smallest AIC is selected.
3. If none of the hypotheses provided a better fit for a time-varying predictor, the full model was chosen.

### Model fit

Model fit was assessed using the concordance (or c) statistic which measures discrimination. This unit-less index denotes “the probability that a randomly selected subject who experienced an outcome will have a higher predicted probability of having the outcome occur compared to a randomly selected subject who did not experience the event”.[35] This index ranges from 0.5 to 1; a reasonable model has a c at least 0.7 while a strong model has a c exceeding 0.8.[36] C-statistics of cross validated models and bootstrapped standard errors were very similar to the initial results (S3 and S4 Files; S2 Table).

### Ethics

This study was approved by the Health Research Ethics Board at the University of Manitoba (#H2013:164) and the Health Information Privacy Commission at Manitoba Health, Seniors and Active Living (#2013/2014-04). Using de-identified administrative data files did not require informed consent from participants.

## Results

Almost 25 percent of the individuals in the cohort did not graduate from high school (24.10%). Table 1 provides descriptive statistics of all variables included in the model; differences between those graduating from high school and those not graduating are identified by *χ*^2^(for binary variables) and t (for continuous variables) statistics. Individuals not graduating from high school differed significantly from those who did on almost all covariates. S3 and S4 Tables presents the correlations among the time-invariant and time-varying measures.

**Table 1:** Descriptive statistics (N = 89,763).

Covariates				Did not Graduate from High School (n = 21,635)	Graduated from High School (n = 68,128)
**Time–Invariant**				**n (%)/ mean(SD)**	**n (%)/ mean(SD)**
Mean Mother's Age at First Birth				23.65 (5.55)	26.98 (5.04)^a^
Mean Family Size (1–5+)				2.85 (1.31)	2.31 (0.94)^a^
Mean Birth Order (1–5+)				1.58 (0.84)	1,46 (0.69)^a^
Born in Rural Neighborhood				12,265 (56.69)	30,268 (44.43)^a^
Mother Unmarried at Time of Birth				11,163 (51.60)	15,610 (22.91)^a^
Male				12,249 (56.62)	33,061 (48.53)^a^
Birth Weight < = 2500g				1,033 (4.77)	3,094 (4.54)
2500g < Birth Weight < = 3500g				10,448 (48.29)	33,263 (48.42)
Birth Weight > 3500g				10,154 (46.93)	31,771 (46.63)
Birth Year				1988 (3.83)	1988 (3.87)
Lower than Average Grade 9 Achievement				20,161 (93.19)	28,883 (42.40)^a^
**Time-Varying**					
	Ages 0–3	Ages 4–8	Ages 9–13		
Lived in Low Income Neighborhood	0	0	0	6,987 (32.29)	45,634 (66.98)^a^
	1	0	0	1,392 (6.43)	4,603 (6.76)^a^
	0	1	0	617 (2.85)	1,567 (2.30)^a^
	0	0	1	1,082 (5.00)	2,438 (3.58)^a^
	1	1	0	1,810 (8.37)	3,912 (5.74)^a^
	1	0	1	569 (2.63)	828 (1.22)^a^
	0	1	1	1,417 (6.55)	2,548 (3.74)^a^
	1	1	1	7,761 (35.87)	6,598 (9.68)^a^
Residential Mobility	0	0	0	5,487 (25.36)	25,575 (37.54)^a^
	1	0	0	1,922 (8.88)	9,341 (13.71)^a^
	0	1	0	2,056 (9.50)	9,639 (14.15)^a^
	0	0	1	1,538 (7.11)	5,438 (8.03)^a^
	1	1	0	2,441 (11.28)	5,714 (8.39)^a^
	1	0	1	1,198 (5.54)	3,553 (5.22)^a^
	0	1	1	2,186 (10.10)	3,769 (5.53)^a^
	1	1	1	4,807 (22.22)	5,069 (7.44)^a^
Family Structure Change	0	0	0	15,215 (70.33)	55,273 (81.13)^a^
	1	0	0	1,891 (8.74)	3,966 (5.82)^a^
	0	1	0	1,988 (9.19)	4,078 (5.99)^a^
	0	0	1	1,502 (6.94)	3,462 (5.08)^a^
	1	1	0	373 (1.72)	537 (0.79)^a^
	1	0	1	308 (1.42)	418 (0.61)^a^
	0	1	1	310 (1.43)	338 (0.50)^a^
	1	1	1	48 (0.22)	57 (0.08)^a^
Externalizing Mental Health	0	0	0	19,599 (90.59)	65,029 (95.45)^a^
	1	0	0	166 (0.77)	445 (0.65)
	0	1	0	321 (1.48)	595 (0.87)^a^
	0	0	1	998 (4.61)	1436 (2.11)^a^
	1	1	0	14 (0.06)	47 (0.07)^a^
	1	0	1	29 (0.13)	17 (0.02)^a^
	0	1	1	484 (2.24)	531 (0.78)^a^
	1	1	1	24 (0.11)	28 (0.04)^a^
Injuries	0	0	0	18,911 (87.41)	63,780 93.62)^a^
	1	0	0	837 (3.87)	1,246 (1.83)^a^
	0	1	0	806 (3.73)	1,439 (2.11)^a^
	0	0	1	887 (4.10)	1,495 (2.19)^a^
	1	1	0	58 (0.27)	53 (0.08)^a^
	1	0	1	59 (0.27)	44 (0.06)^a^
	0	1	1	66 (0.31)	64 (0.09)^a^
	1	1	1	11 (0.05)	7(0.01) ^a^

^a^ difference between those graduating and those not graduating from high school significant at < 0.05

### Model selection

In the final model, the temporal pattern selected differed among the time-varying predictors. S1 Table details the model selection process. The ‘Full’ model best described the temporal pattern of living in a low-income neighborhood, residential mobility, and family structure change. This model indicates that all time periods are important but different trajectories over childhood and adolescence have different effects on high school graduation. Externalizing mental conditions were best approached using a ‘Sensitive Periods’ model, while the risk associated with injuries was best described by the ‘Accumulation of Risk’ framework. The final models included the temporal pattern selected for each time-varying predictor and adjusted for time-invariant predictors. The full (with 45 predictors) and final (more parsimonious) models showed excellent discrimination, both having c-statistics of 0.859.

### Model including all variables

Table 2 highlights the temporal pattern of events associated with failure to graduate from high school and the time-invariant predictors included for adjustment. Being born to an unmarried mother was associated with failure to graduate. Living in a low-income neighborhood and residential mobility at any point are associated with higher odds of failure to graduate. These measures were particularly important in the period prior to the outcome. Externalizing mental health conditions impact high school graduation most when the diagnoses occurred in the age group prior to the outcome. Individuals with more frequent injuries had significantly higher odds of failing to graduate high school. The variable most strongly increasing the odds of failing to graduate from high school was having less than average grade 9 achievement, followed by living in a low-income neighborhood in all age groups and by residential mobility in all age groups.

**Table 2:** Odds ratios for failure to graduate from high school with time-invariant covariates.

Covariates	OR	95% CI
**Time-Invariant**		
Mother's Age at First Birth	0.98	0.97–0.98
Family Size	1.28	1.26–1.31
Birth Order	1.13	1.10–1.16
Rural	1.32	1.27–1.38
Mother Unmarried at Time of Birth	2.03	1.94–2.12
Male	1.17	1.12–1.21
Birth Weight < = 2500g	0.94	0.86–1.03
Birth Weight > 3500g	0.99	0.95–1.03
Birth Year	0.94	0.93–0.94
Lower than Average Grade 9 Achievement	11.71	11.05–12.39
**Time-Varying**		
Low Income Neighborhood, 100	1.36	1.26–1.47
Low Income Neighborhood, 010	1.52	1.35–1.70
Low Income Neighborhood, 001	1.70	1.56–1.86
Low Income Neighborhood, 110	1.69	1.57–1.82
Low Income Neighborhood, 101	2.07	1.82–2.36
Low Income Neighborhood, 011	1.86	1.72–2.02
Low Income Neighborhood, 111	3.21	3.05–3.37
Residential Mobility, 100	1.05	1.00–1.14
Residential Mobility, 010	1.07	1.00–1.14
Residential Mobility, 001	1.23	1.14–1.33
Residential Mobility, 110	1.33	1.24–1.42
Residential Mobility, 101	1.18	1.08–1.28
Residential Mobility, 011	1.61	1.50–1.74
Residential Mobility, 111	1.68	1.58–1.80
Family Structure Change, 100	1.03	0.96–1.11
Family Structure Change, 010	1.25	1.16–1.36
Family Structure Change, 001	1.26	1.17–1.36
Family Structure Change, 110	0.93	0.80–1.10
Family Structure Change, 101	1.03	0.86–1.22
Family Structure Change, 011	1.35	1.12–1.63
Family Structure Change, 111	1.16	0.74–1.81
Externalizing Mental Health Condition, 0–3	1.26	1.05–1.53
Externalizing Mental Health Condition, 4–8	1.28	1.14–1.44
Externalizing Mental Health Condition, 9–13	1.60	1.47–1.75
Number of Time Periods with Injuries	1.33	1.26–1.41
C-Statistic	0.859	

Note: Time varying predictors are included based on model selection in Table 3; full model was selected for ‘Low Income Neighborhood’, ‘Residential Mobility’, ‘Family Structure Changes’; sensitive period model was selected for ‘Externalizing Mental Health Conditions’, and the accumulation of risk model was selected for ‘Injuries’

### Additional models

We have controlled for a series of time-invariant background measures in modelling the temporal pattern which best describes the influence of each time-varying predictor. To determine whether model selection differed without these background variables, several additional strategies were examined: a) a model controlling only for covariates at birth (this excludes the grade 9 achievement variable) (selected model identified by ○ in Table 3 and in Table 4; c-statistic = 0.790); b) a model that only included the time-varying predictors, not controlling for any of the background variables (selected model identified by ◆ in Table 3 and in Table 5; c-statistic = 0.734).

**Table 3:** Difference in temporal pattern selected when adjusting for different confounders.

Time-Varying Predictor	Full	Accumulation of Risk	Sensitive Periods	Critical Period		
				Early Childhood (0–3 years)	Early Elementary (4–8 years)	Early Adolescence (9–13 years)
Low Income Neighborhood	●○◆∎					
Residential Mobility	●○◆∎					
Family Structure Changes	●○◆∎					
Externalizing Mental Conditions	●○●	◆				
Injuries		●○∎	◆			

● with all time-invariant covariates ○ with time-invariant birth covariates (excluding grade 9 achievement)

◆ without time-invariant covariates ∎ sibling analysis (with all time-invariant covariates)

**Table 4:** Odds ratios for failure to graduate from high school with time-invariant covariates (excluding grade 9 achievement).

Covariates	OR	95% CI
**Time-Invariant**		
Mother's Age at First Birth	0.96	0.96–0.97
Family Size	1.32	1.30–1.34
Birth Order	1.17	1.15–1.20
Rural	1.41	1.36–1.47
Mother Unmarried at Time of Birth	2.45	2.35–2.56
Male	1.49	1.44–1.54
Birth Weight < = 2500g	1.01	0.93–1.10
Birth Weight > 3500g	0.97	0.93–1.01
Birth Year	0.94	0.93–0.94
**Time-Varying**		
Low Income Neighborhood, 100	1.45	1.45–1.35
Low Income Neighborhood, 010	1.64	1.47–1.82
Low Income Neighborhood, 001	1.92	1.77–2.08
Low Income Neighborhood, 110	1.90	1.77–2.03
Low Income Neighborhood, 101	2.44	2.16–2.75
Low Income Neighborhood, 011	2.20	2.04–2.38
Low Income Neighborhood, 111	3.95	3.77–4.14
Residential Mobility, 100	1.04	0.98–1.11
Residential Mobility, 010	1.07	1.00–1.13
Residential Mobility, 001	1.24	1.16–1.34
Residential Mobility, 110	1.39	1.30–1.49
Residential Mobility, 101	1.28	1.18–1.39
Residential Mobility, 011	1.73	1.61–1.86
Residential Mobility, 111	1.88	1.77–2.00
Family Structure Change, 100	1.08	1.01–1.16
Family Structure Change, 010	1.37	1.28–1.46
Family Structure Change, 001	1.42	1.32–1.53
Family Structure Change, 110	0.99	0.85–1.15
Family Structure Change, 101	1.04	0.88–1.23
Family Structure Change, 011	1.43	1.20–1.71
Family Structure Change, 111	1.22	0.80–1.87
Externalizing Mental Health Condition, 100	1.31	1.08–1.61
Externalizing Mental Health Condition, 010	1.64	1.40–1.92
Externalizing Mental Health Condition, 001	2.37	2.15–2.60
Externalizing Mental Health Condition, 110	0.84	0.44–1.62
Externalizing Mental Health Condition, 101	5.13	2.63–9.98
Externalizing Mental Health Condition, 011	3.02	1.35–4.71
Externalizing Mental Health Condition, 111	2.52	1.37–1.53
Number of Time Periods with Injuries	1.45	
C-Statistic	0.790	

Note: Time varying predictors are included based on model selection in Table 3; full model was selected for ‘Low Income Neighborhood’, ‘Residential Mobility’, ‘Family Structure Changes’, and ‘Externalizing Mental Health Conditions’, sensitive period model was selected for ‘Injuries’

**Table 5:** Odds ratios for failure to graduate from high school without time-invariant covariates.

Covariates	OR	95% CI
**Time-Varying**		
Low Income Neighborhood, 100	1.65	1.54–1.76
Low Income Neighborhood, 010	1.93	1.74–2.13
Low Income Neighborhood, 001	2.33	2.15–2.52
Low Income Neighborhood, 110	2.30	2.16–2.45
Low Income Neighborhood, 101	3.37	3.01–3.78
Low Income Neighborhood, 011	2.84	2.64–3.05
Low Income Neighborhood, 111	6.23	5.96–6.50
Residential Mobility, 100	0.96	0.91–1.02
Residential Mobility, 010	1.05	0.99–1.12
Residential Mobility, 001	1.24	1.15–1.32
Residential Mobility, 110	1.54	1.44–1.63
Residential Mobility, 101	1.24	1.15–1.34
Residential Mobility, 011	2.01	1.88–2.15
Residential Mobility, 111	2.24	2.12–2.37
Family Structure Change, 100	1.36	1.27–1.45
Family Structure Change, 010	1.33	1.25–1.42
Family Structure Change, 001	1.27	1.18–1.36
Family Structure Change, 110	1.40	1.20–1.62
Family Structure Change, 101	1.53	1.30–1.80
Family Structure Change, 011	1.73	1.46–2.05
Family Structure Change, 111	1.44	0.95–2.19
Number of Time Periods with Externalizing Mental Diagnoses	1.73	1.65–1.82
Injuries, 0–3	1.80	1.64–1.98
Injuries, 4–8	1.62	1.48–1.78
Injuries, 9–13	1.80	1.65–1.96
C-Statistic	0.734	

Note: Time varying predictors are included based on model selection in Table 3; full model was selected for ‘Low Income Neighborhood’, ‘Residential Mobility’, and ‘Family Structure Changes’, accumulation of risk model was selected for ‘Externalizing Mental Health Conditions’, sensitive period model was selected for ‘Injuries’

Without including Grade 9 achievement, the externalizing mental health conditions—particularly those diagnosed in the 9–13 age period—show dramatically higher odds ratios (Table 4). Compared with Table 2, somewhat higher odds ratios are seen for those living in a low income neighborhood and experiencing residential mobility. Table 5 demonstrates the effects of removing all the time invariant measures. The importance of income is highlighted. The role of the externalizing mental health conditions changes, with the accumulation of risk model providing a better fit.

Omitted variable bias is a common concern in multi-variate analysis.[5,18,37] Sibling analyses address this by capitalizing on common characteristics within the family; our fourth analysis included all time-invariant covariates in a sample incorporating children having at least one sibling in the cohort (n = 102, 869). A multilevel logistic regression model accounted for unmeasured shared family-level characteristics (selected model identified by ∎ in Table 3 and in S5 Table; c-statistic = 0.866). Results were generally similar to those in the other models, indicating that omitted variable bias was not a major influence on the results.

Table 3 highlights consistency (social variables)–and some differences (health variables)–among the strategies; excluding time invariant covariates significantly decreased model fit. Temporal patterns associated with externalizing mental health conditions became more important, but the confidence intervals of many of the odds ratios are wide.

## Discussion

The temporal patterns by which time-varying predictors affected high school graduation differed among predictors. Tables 2–5 have shown that predictors including the event in early adolescence generated the highest odds ratios. Others have suggested that baseline and early childhood measures tend to become less predictive of late adolescence and adult outcomes.[37] This paper also highlights the transmission of risk across developmental periods; exposure to adversity in one developmental period had significant implications for adversity in subsequent life stages.[38]

These Canadian results differ somewhat from American findings based on the long-running Panel Study of Income Dynamics (PSID). Although some PSID analyses regarding education were ambiguous, “family economic conditions in early childhood appear to matter more for shaping later development than economic conditions during adolescence.”[39] Methodological differences between approaches might contribute to differences in results. Thus, Duncan et al. used a much smaller sample (effective N = 1,254), incorporating various time invariant measures (including race) and a single time-varying measure, childhood income (the average annual household income in three periods—prenatal to age 5, ages 6–10, and ages 11–15).[39]

Additionally, Canadian-American differences in child development may build on differences in inequality between Canada and the United States. Inequality in the United States is relatively high compared with other OECD countries; Canadian provinces and metropolitan areas both have lower income inequality than their American counterparts and stronger social programs directed toward this inequality.[40,41]

Previous life-course studies using similar methods have only rarely included time-invariant confounders.[42–46] This raises problems. First, while excluding background variables facilitates distinguishing among model fit for different temporal patterns, failing to include important covariates is likely to overestimate the association between the exposures and outcome.[47] Second, including background variables may change the temporal pattern determined to be the best fit for the time-varying predictor. As seen in Tables 2–5, the models selected differ somewhat when controlling for background variables. The very good fit of our models suggests that generally ‘correct’ temporal patterns are identified. Given our analyses of omitted variable bias, unavailable measures (such as parental education and race) seem unlikely to explain a great deal more variance, although ‘aboriginal status’ would probably be a significant time-invariant measure.

Which confounders should be controlled for in life-stage modelling? Our models include several measures of circumstance at birth and one at early adolescence (the Grade 9 Achievement Index). The Grade 9 Achievement Index is by far the strongest predictor of high school graduation and probably “soaks up” at least part of the impact of measures from earlier ages. Tables 2–5 and S5 Table show that strong associations between age category and educational attainment remain with and without this index.

In analyses excluding grade 9 achievement, externalizing mental health conditions expressed in ages 9–13 were very important predictors. Income in the neighborhood of residence consistently showed high correlations with such income in an earlier or later period. Despite these relationships, the odds ratios associated with different income/neighborhood combinations varied substantially. Residence in such low-income neighborhoods over all three time periods showed strong, regular relationships. In other circumstances, the temporal patterns selected for the time-varying covariates might vary, given the complexities of life course epidemiology.[48]

Administrative data provide major advantages, both generally and specifically for life course research. The very large number of cases allows examining variables in considerable detail across age groups. In contrast, cohort studies (such as PSID) using a much smaller N typically rely on high cost surveys. This smaller N results in lower precision when such techniques as sibling difference models (capitalizing on within family variations) are used to deal with such bias. This is important; adjustments for omitted variable bias sometimes produce large reductions in the estimated effects of income. (37) Surveys can be unreliable, may suffer from higher nonresponse rates and report events “as occurring more recently than they did in fact occur”.[37] Administrative data both allow more accurate timing of events (such as diagnosis and family structure changes) essential for assessing risk in different age groups and facilitate the inclusion of important confounders.[9]

Administrative data clearly have limitations. Research using such data often has to be opportunistic in relying on (or not relying on) certain variables. Checks on unavailable variables can help; pilot studies, different statistical approaches, and comparisons with other work are often feasible.[21] Specific study limitations include the use of binary exposure and outcome variables for simplification (but the models are already complicated). Symptoms may occur well before an official diagnosis and family instability often precedes a formal change in family structure (such as divorce or remarriage). We defined externalizing mental health diagnoses as at least two ADHD and/or CD/ODD diagnoses in a four-year time period. Defining this variable differently may change its “effect” on high school graduation. However, linkable data bases allow ‘expansion’ through the creation of new variables. Over time, additional measures (such as child maltreatment, parental welfare use, prescription use, and maternal education) will facilitate long-term cohort research and might conceivably change model selection. Analyses of temporal patterns can be useful in studying such other outcomes as educational achievement, teen pregnancy, and workforce participation.

This research presents an important way to build on the strengths of large population-based data. Complicated time-dependent models are dependent on such data sets. The alternative seems to rely exclusively on simpler models based on having more information per person, but many fewer cases. Temporal patterns have significant implications on the timing of the best intervention. An exposure important at a specific period of time (critical period) suggests an early intervention.[7] Exposures operating in sensitive periods warrant intervention at “strategic junctures in the life course”, whereas exposures accumulating over time require interventions “consistently over time, building on models of social change”.[7] Because exposures operate through different processes, the timing of an intervention for a specific exposure may vary.

The relationships among several measures of social behavior, cognitive development, and health have been explored.[9] Life course models that best fit each time-varying predictor have been identified for one geographic area; replication elsewhere is called for. A stronger focus on adolescence as a “second sensitive developmental period” appears warranted. The rapid brain maturation associated with early adolescence can significantly modify childhood trajectories. The powerful and consistent role of income has been established. Efforts to support families remaining in the same homes or in the same schools (despite residential mobility) seem appropriate.

## Supporting information

S1 File Data quality. (PDF)

S2 File Cohort selection. (PDF)

S3 File Cross validation. (PDF)

S4 File Bootstrapping. (PDF)

S1 Fig Cohort selection. (TIF)

S1 Table P-values of likelihood ratio tests against full models and AIC (Selected models are in bold). (PDF)

S2 Table Bootstrapped standard errors. (PDF)

S3 Table Pearson Correlation Coefficients for time-invariant covariates. (PDF)

S4 Table Pearson Correlation Coefficients for time-varying covariates. (PDF)

S5 Table Odds ratios for failure to graduate from high school, cohort B. (PDF)
