# Supplementary material for: Life course epidemiology: Modeling educational attainment with administrative data
Source: PLoS One. 2017 Dec 27;12(12):e0188976. doi: 10.1371/journal.pone.0188976 (PMC5744927; doi:10.1371/journal.pone.0188976)
Supplement: S1 File — (PDF) [file pone.0188976.s001.pdf]

S1 File. Data quality and loss to follow-up

As military personnel and Royal Canadian Mounted Police personnel and their families are insured federally, they are not included in the registry; over 98% of births noted by Vital Statistics are in the registry. An individual Personal Health Identification Number (PHIN) (since 1984) specifies each person; these PHINs are subsequently scrambled to de-identify the individuals involved. Each substantive file is checked against the registry for accuracy of the identifiers and for such information as date of in-hospital death. Children are linked to mothers using hospital birth record information; the mother was noted in essentially all cases and fathers are specified in approximately 80% of cases.[1] Several checks applied to the birth cohorts (looking at missing data, the number of children designated as having the same mother and father, and complicated blended families) have confirmed the accuracy of the algorithm. An attrition rate of approximately 23%, primarily due to migration out-of-province in the original cohort, was largely uncorrelated with several measures of health and socioeconomic status. After controlling for family fixed effects, estimates of the impact of infant health on later outcomes do not appear to be biased from out of province mobility.[2] Childhood deaths provide relatively little attrition; children dying before age eight were much less healthy at birth with most deaths occurring early in life.[1]

References

1. Currie J, Stabile M, Manivong P, Roos L. Child health and young adult outcomes. J Hum Resour. 2010;4: 517–548.
2. Oreopoulos P, Stabile M, Walld R, Roos L. Short, medium, and long term consequences of poor infant health: An analysis using siblings and twins. J Hum Resour. 2008;43: 88–138.
