# Supplementary material for: Life course epidemiology: Modeling educational attainment with administrative data
Source: PLoS One. 2017 Dec 27;12(12):e0188976. doi: 10.1371/journal.pone.0188976 (PMC5744927; doi:10.1371/journal.pone.0188976)
Supplement: S2 File — (PDF) [file pone.0188976.s002.pdf]

S2 File – Cohort Selection

Individuals with intellectual disability were excluded. Individuals with missing data on: birth weight, birth order, family structure history, mother's age at first birth, family identifier, high school graduation, socioeconomic status of the neighborhood at birth, child and family services variables, parental use of income assistance, and an index of grade 9 achievement were not included. The variable with the most missing data was high school graduation status. Of the 10,866 individuals who were missing information on high school graduation, 71% lived in rural Manitoba. Many schools in rural Manitoba (particularly in the north) are run by First Nations communities, which are not required to report information to the Ministry of Education and Training. Analyses assuming "no high school graduation" for this group produced similar results.

The primary cohort (Cohort A) included one child per family; simple random sampling without replacement is used to select this child (S1 Fig). An additional analysis used a siblings sample, which consisted of families having at least two children in the cohort (Cohort B).
