# Supplementary material for: Life course epidemiology: Modeling educational attainment with administrative data
Source: PLoS One. 2017 Dec 27;12(12):e0188976. doi: 10.1371/journal.pone.0188976 (PMC5744927; doi:10.1371/journal.pone.0188976)
Supplement: S3 File — (PDF) [file pone.0188976.s003.pdf]

S3 File. Cross validation

To determine that our models were not over-fit, we used 10-fold cross validation. The following steps were used to cross validate our sample:

1. Randomly divide your data into 10 pieces, 1 through k.

2. Treat the 1st tenth of the data as the test dataset. Fit the model to the other nine-tenths of the data (which are now the training data).

3. Apply the model to the test data (e.g., for logistic regression, calculate predicted probabilities of the test observations).

4. Repeat this procedure for all 10 tenths of the data.

5. Calculate statistics of model accuracy and fit (e.g., ROC curves) from the test data only.[1]

The c-statistic was calculated as the measure of fit. The C-statistic for the final model (as presented in the paper) was 0.859 (95% CI 0.856-0.862); the c-statistics obtained after cross-validation was 0.858 (95% CI 0.856-0.861). The fit statistics were not significantly different, indicating that our models were robust.

References

1. Sainani K. Bootstrapping and Cross Validation [Power Point] [Internet]. 2013 [cited 30 Sep 2014]. Available: web.stanford.edu/~kcobb/hrp261/lecture7.ppt
