# Supplementary material for: Life course epidemiology: Modeling educational attainment with administrative data
Source: PLoS One. 2017 Dec 27;12(12):e0188976. doi: 10.1371/journal.pone.0188976 (PMC5744927; doi:10.1371/journal.pone.0188976)
Supplement: S4 File — (PDF) [file pone.0188976.s004.pdf]

S4 File. Bootstrapping

As an additional test to determine the robustness of our results, bootstrapping was done to determine the standard errors associated with our estimates. Unrestricted random sampling with replacement was done at the individual level; each outcome was modelled 500 times with different randomly selected samples. This analysis produced results very similar to the initial analysis (S2 Table).
