# Supplementary figures and images for: Life course epidemiology: Modeling educational attainment with administrative data
Source: PLoS One. 2017 Dec 27;12(12):e0188976. doi: 10.1371/journal.pone.0188976 (PMC5744927; doi:10.1371/journal.pone.0188976)

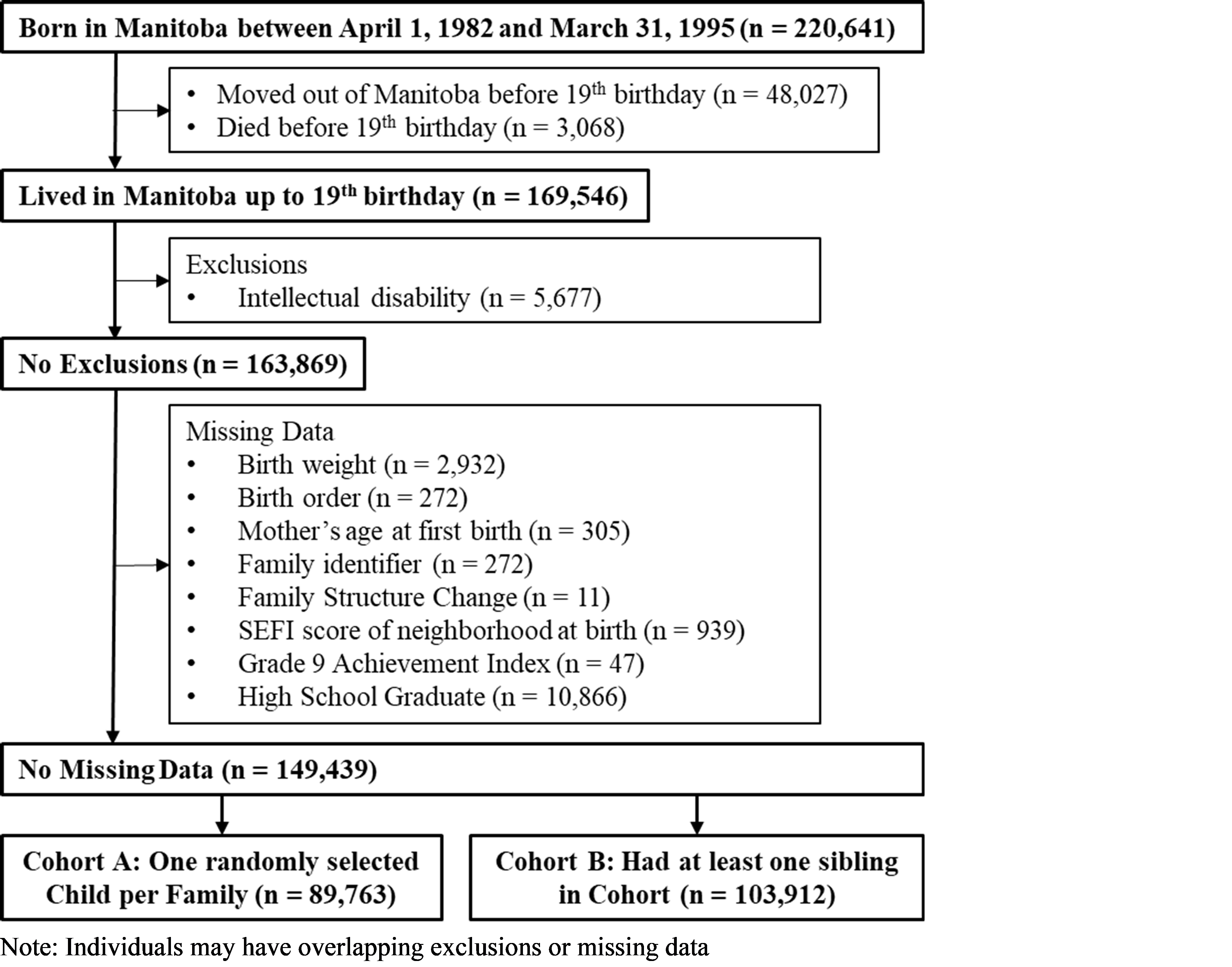

Supplement: S1 Fig — (TIF) [file pone.0188976.s005.tif]
