# Supplementary material for: Life course epidemiology: Modeling educational attainment with administrative data
Source: PLoS One. 2017 Dec 27;12(12):e0188976. doi: 10.1371/journal.pone.0188976 (PMC5744927; doi:10.1371/journal.pone.0188976)
Supplement: S1 Table — (PDF) [file pone.0188976.s006.pdf]

S1 Table. P-values of likelihood ratio tests against full models and AIC (Selected models are in bold)

				Critical Period		
**Time-Varying Predictor**	Statistic	Accumulation of Risk	Sensitive Periods	Early Childhood (0-3 years)	Early Elementary (4 - 8 years)	Early Adolescence (9-13 years)
Low Income Neighborhood	LRT P-Value	<0.01	<0.01	<0.01	<0.01	<0.01
	AIC	69549.66	69519.09	70387.88	70143.79	70016.04
Residential Mobility	LRT P-Value	<0.01	<0.01	<0.01	<0.01	<0.01
	AIC	69559.64	69521.27	69824.42	69668.28	69657.65
Family Structure Changes	LRT P-Value	<0.01	<0.01	<0.01	<0.01	<0.01
	AIC	69520.95	69497.19	69554.93	69527.43	69521.76
Externalizing Mental Conditions	LRT P-Value	0.02	**0.05**	<0.01	<0.01	<0.01
	AIC	69493.53	**69491.39**	69647.83	69590.91	69512.09
Injuries	LRT P-Value	**0.45**	0.72	<0.01	<0.01	<0.01
	AIC	**69483.85**	69484.21	69569.52	69570.48	69535.94

Note: Model Selection Reason: If more than one nested model had a p-value greater than 0.05, then the model with the smallest AIC was selected. If no alternative models are bolded, the full model is selected.
