# Supplementary material for: Life course epidemiology: Modeling educational attainment with administrative data
Source: PLoS One. 2017 Dec 27;12(12):e0188976. doi: 10.1371/journal.pone.0188976 (PMC5744927; doi:10.1371/journal.pone.0188976)
Supplement: S2 Table — (PDF) [file pone.0188976.s007.pdf]

S2 Table. Bootstrapped Standard Errors

	OR	Model 95% CI	Bootstrap 95% CI
**Time-Invariant Predictors**			
Mother's Age at First Birth	0.98	0.97-0.98	0.98-0.98
Family Size	1.28	1.26-1.31	1.26-1.31
Birth Order	1.13	1.10-1.16	1.10-1.16
Rural	1.32	1.27-1.38	1.27-1.38
Mother Unmarried at Time of Birth	2.03	1.94-2.12	1.94-2.12
Male	1.17	1.12-1.21	1.12-1.22
Birth Weight <= 2500g	0.94	0.86-1.03	0.86-1.03
Birth Weight > 3500g	0.99	0.95-1.03	0.95-1.03
Birth Year	0.94	0.93-0.94	0.93-0.95
Lower than Average Grade 9 Achievement	11.71	11.05-12.39	11.06-12.39
**Time-Varying Predictor**			
Low Income Neighborhood, 100	1.36	1.26-1.47	1.25-1.47
Low Income Neighborhood, 010	1.52	1.35-1.70	1.36-1.70
Low Income Neighborhood, 001	1.70	1.56-1.86	1.55-1.86
Low Income Neighborhood, 110	1.69	1.57-1.82	1.56-1.83
Low Income Neighborhood, 101	2.07	1.82-2.36	1.81-2.37
Low Income Neighborhood, 011	1.86	1.72-2.02	1.71-2.02
Low Income Neighborhood, 111	3.21	3.05-3.37	3.05-3.38
Residential Mobility, 100	1.05	1.00-1.14	0.98-1.12
Residential Mobility, 010	1.07	1.00-1.14	1.00-1.15
Residential Mobility, 001	1.23	1.14-1.33	1.14-1.33
Residential Mobility, 110	1.33	1.24-1.42	1.23-1.43
Residential Mobility, 101	1.18	1.08-1.28	1.08-1.29
Residential Mobility, 011	1.61	1.50-1.74	1.49-1.74
Residential Mobility, 111	1.68	1.58-1.80	1.57-1.80
Family Structure Change, 100	1.03	0.96-1.11	0.96-1.11
Family Structure Change, 010	1.25	1.16-1.36	1.16-1.34
Family Structure Change, 001	1.26	1.17-1.36	1.16-1.36
Family Structure Change, 110	0.93	0.80-1.10	0.79-1.10
Family Structure Change, 101	1.03	0.86-1.22	0.85-1.25
Family Structure Change, 011	1.35	1.12-1.63	1.11-1.64
Family Structure Change, 111	1.16	0.74-1.81	0.71-1.90
Externalizing Mental Health Condition, 0 – 3	1.26	1.05-1.53	1.02-1.55
Externalizing Mental Health Condition, 4 – 8	1.28	1.14-1.44	1.14-1.44
Externalizing Mental Health Condition, 9 - 13	1.60	1.47-1.75	1.46-1.75
Number of Time Periods with Injuries	1.33	1.26-1.41	1.25-1.42
