# Supplementary material for: Life course epidemiology: Modeling educational attainment with administrative data
Source: PLoS One. 2017 Dec 27;12(12):e0188976. doi: 10.1371/journal.pone.0188976 (PMC5744927; doi:10.1371/journal.pone.0188976)
Supplement: S3 Table — (PDF) [file pone.0188976.s008.pdf]

S3 Table. Pearson Correlation Coefficients for Time-Invariant Covariates

	Mean Family Size (1-5+)	Mean Birth Order (1-5+)	Born in Rural Neighborhood	Mother Unmarried at Time of Birth	Male	Birth Weight <= 2500g	2500g < Birth Weight <= 3500g	Birth Weight > 3500g	Birth Year	Lower than Average Grade 9 Achievement
Mean Mother's Age at First Birth	-0.34	-0.20	-0.09	-0.29	0.004	0.03	0.01	-0.02	-0.03	-0.23
Mean Family Size (1-5+)		0.32	0.10	0.09	-0.01	-0.02	-0.04	0.05	0.17	0.13
Mean Birth Order (1-5+)			0.03	-0.13	0.004	-0.03	-0.05	0.07	0.06	0.05
Born in Rural Neighborhood				-0.02	-0.001	-0.01	-0.02	0.03	-0.25	0.08
Mother Unmarried at Time of Birth					-0.01	0.02	0.02	-0.03	0.22	0.21
Male						-0.02	-0.11	0.11	-0.01	0.15
Birth Weight <= 2500g							-0.21	-0.21	0.01	0.02
2500g < Birth Weight <= 3500g								-0.91	0.002	-0.006
Birth Weight > 3500g									-0.01	-0.001
Birth Year										-0.01
