# Supplementary material for: Life course epidemiology: Modeling educational attainment with administrative data
Source: PLoS One. 2017 Dec 27;12(12):e0188976. doi: 10.1371/journal.pone.0188976 (PMC5744927; doi:10.1371/journal.pone.0188976)
Supplement: S4 Table — (PDF) [file pone.0188976.s009.pdf]

S4 Table. Pearson Correlation Coefficients for Time-Varying Covariates

		Lived in Low Income Neighborhood			Residential Mobility			Family Structure Change			Externalizing Mental Health			Injuries		
		0 - 3	4 - 8	9 - 13	0 - 3	4 - 8	9 - 13	0 - 3	4 - 8	9 - 13	0 - 3	4 - 8	9 - 13	0 - 3	4 - 8	9 - 13
Lived in Low Income Neighborhood	0 - 3		0.64	0.48	0.26	0.18	0.16	0.08	0.05	0.02	0.002	0.02	0.02	0.05	0.03	0.03
	4 - 8			0.64	0.12	0.34	0.17	0.07	0.08	0.03	-0.0003	0.02	0.01	0.05	0.04	0.03
	9 - 13				0.10	0.11	0.22	0.06	0.05	0.06	-0.01	0.01	0.01	0.06	0.04	0.04
Residential Mobility	0 - 3					0.21	0.21	0.15	0.09	0.05	0.02	0.05	0.06	0.005	0.002	0.001
	4 - 8						0.24	0.09	0.19	0.07	0.02	0.05	0.05	0.02	0.01	0.004
	9 - 13							0.08	0.11	0.19	0.02	0.05	0.05	0.02	0.01	0.02
Family Structure Change	0 - 3								0.05	0.04	0.005	0.02	0.03	0.01	0.01	0.01
	4 - 8									0.03	0.01	0.03	0.04	0.01	0.004	0.001
	9 - 13										0.004	0.02	0.03	0.01	0.01	0.01
Externalizing Mental Health	0 - 3											0.08	0.04	0.0001	0.0004	0.005
	4 - 8												0.38	0.01	0.003	0.003
	9 - 13													0.01	0.01	0.02
Injuries	0 - 3														0.03	0.02
	4 - 8															0.03
	9 - 13															

Note: All variables used here are dichotomous
