# Supplementary material for: Life course epidemiology: Modeling educational attainment with administrative data
Source: PLoS One. 2017 Dec 27;12(12):e0188976. doi: 10.1371/journal.pone.0188976 (PMC5744927; doi:10.1371/journal.pone.0188976)
Supplement: S5 Table — (PDF) [file pone.0188976.s010.pdf]

S5 Table. Odds Ratios for Failure to Graduate from High School, Cohort B

Covariates	OR	95% CI
**Time-Invariant**		
Mother's Age at First Birth	0.96	0.95-0.96
Family Size	1.60	1.56-1.65
Birth Order	1.15	1.12-1.2
Rural	1.35	1.28-1.42
Mother Unmarried at Time of Birth	2.49	2.34-2.64
Male	1.31	1.25-1.37
Birth Weight <= 2500g	0.95	0.85-1.06
Birth Weight > 3500g	0.92	0.88-0.97
Birth Year	0.96	0.95-0.96
Less than Average Grade 9 Achievement	15.03	14.06-16.06
**Time-Varying**		
Low Income Neighborhood, 100	1.57	1.43-1.73
Low Income Neighborhood, 010	1.70	1.46-1.97
Low Income Neighborhood, 001	1.89	1.69-2.12
Low Income Neighborhood, 110	2.13	1.94-2.35
Low Income Neighborhood, 101	2.74	2.33-3.23
Low Income Neighborhood, 011	2.60	2.33-2.9
Low Income Neighborhood, 111	4.60	4.28-4.94
Residential Mobility, 100	1.08	0.99-1.17
Residential Mobility, 010	1.17	1.08-1.27
Residential Mobility, 001	1.25	1.14-1.38
Residential Mobility, 110	1.53	1.4-1.67
Residential Mobility, 101	1.23	1.1-1.37
Residential Mobility, 011	1.88	1.7-2.08
Residential Mobility, 111	2.16	1.98-2.36
Family Structure Change, 100	1.12	1.02-1.22
Family Structure Change, 010	1.48	1.35-1.63
Family Structure Change, 001	1.58	1.43-1.74
Family Structure Change, 110	1.06	0.86-1.31
Family Structure Change, 101	1.14	0.91-1.43
Family Structure Change, 011	1.27	0.99-1.64
Family Structure Change, 111	1.32	0.76-2.3
Externalizing Mental Health Condition, 100	1.02	0.77-1.35
Externalizing Mental Health Condition, 010	1.53	1.23-1.9
Externalizing Mental Health Condition, 001	2.19	1.93-2.48
Externalizing Mental Health Condition, 110	0.74	0.28-1.93
Externalizing Mental Health Condition, 101	2.32	0.98-5.51
Externalizing Mental Health Condition, 011	2.06	1.68-2.52
Externalizing Mental Health Condition, 111	1.08	0.41-2.85
Number of Time Periods with Injuries	1.37	1.21-1.54

Note 1: Time-varying predictors are included based on model selection in Table 3; the full model was selected for ‘Low Income Neighborhood’, ‘Residential Mobility’, ‘Family Structure Changes’, and ‘Externalizing Mental Health Conditions’, and the accumulation of risk period model was selected for ‘Injuries’

Note 2: Estimates reflect the adjustment of unmeasured shared family-level variables (such as household income and parental education) using a multilevel logistic regression model
